# A potential misdiagnosis in the COVID-19 pandemic era: a case report of superimposed thrombosis or tumor recurrence

**DOI:** 10.1186/s13019-021-01697-3

**Published:** 2021-10-29

**Authors:** Arezou Zoroufian, Reza Mohseni-Badalabadi, Mehrdad Mahalleh, Seyyed Mojtaba Ghorashi, Sharam Momtahen, Negar Omidi

**Affiliations:** Cardiovascular Disease Research Institute, Tehran Heart Center, Tehran University of Medical Sciences, Tehran, Iran

**Keywords:** Liposarcoma, COVID-19, Thrombosis, Case report

## Abstract

**Background:**

The clinical manifestations of coronavirus disease 2019 (COVID-19) overlap with those of other disorders, especially cardiovascular disease.

**Case presentation:**

We herein describe a 58-year-old woman who presented with syncopal episodes and dyspnea on exertion with a left atrial (LA) mass, scheduled for surgical removal and mitral valve replacement. Nearly 3 months later, the patient developed dyspnea, fever, and a sore throat, resulting in hospital admission with suspected COVID-19. During the diagnostic evaluation, a larger LA mass was detected. The mass seemed to be a COVID-19–induced organized thrombus with prosthetic mitral valve malfunction. Resection was, therefore, planned. An immunohistochemistry study revealed a liposarcoma.

**Conclusions:**

The unusual early recurrence of liposarcomas and the misdiagnosis with COVID-19–induced thrombosis are the hallmark of the present case.

## Introduction

The recent pandemic of coronavirus disease 2019 (COVID-19) has become the most challenging issue worldwide. Indeed, more than 40 million cases have already been identified, with the mortality rate exceeding 1 million. The similarities in the presentations of COVID-19 and cardiovascular disease have hindered the diagnosis and management of the latter, particularly in patients with prosthetic heart valves [1, 2].

Primary cardiac sarcomas are rare, aggressive, and lethal. The autopsy incidence rate of primary cardiac neoplasms is extremely low in that they account for approximately 0.2% of all cardiovascular surgical cases. Ninety-five percent of malignant cardiac tumors manifest themselves as cardiac sarcomas [3–5]. The symptoms are generally nonspecific, and they depend on the location and infiltration of the tumor into adherent tissues. The diagnosis is established on the basis of clinical history and multimodality imaging. Surgical removal and radiotherapy serve as a basis for local restriction, accompanied by chemotherapy for systemic disease [3, 6].

We herein describe a middle-aged woman with a left atrial (LA) mass, which was preoperatively suspected as a myxoma. A new LA mass was detected within about 3 months after complete surgical resection, which was suspected as a COVID-19–induced thrombus, finally diagnosed as a dedifferentiated liposarcoma in the postoperative pathological examination. This case highlights the unusual early recurrence of liposarcomas and the misdiagnosis with COVID-19-induced thrombosis.

## Case presentation

**Fig. 1:**
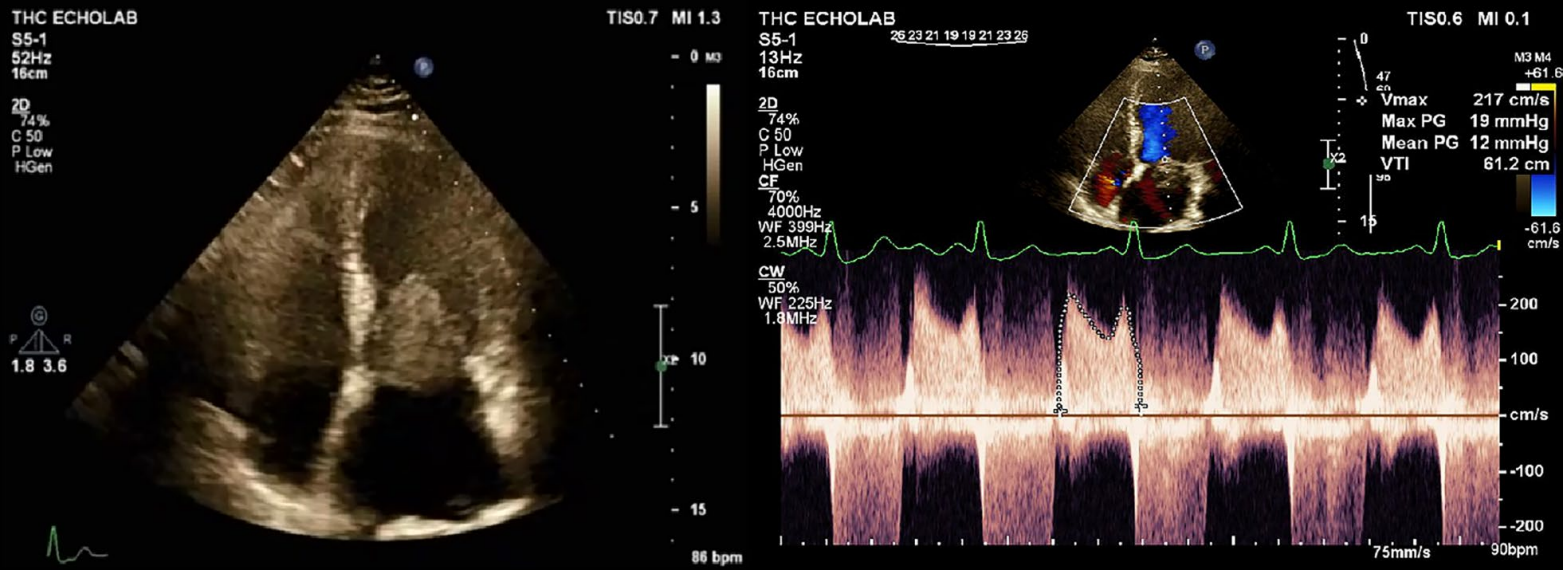
Large round mobile mass attached to tip of anterior mitral valve leaflet (29 × 20 mm) protrude to left ventricle producing significant functional mitral stenosis (apical 4-chamber view in transthoracic echocardiography)

A 58-year-old woman presented with dyspnea on exertion of 2 months’ duration and 2 episodes of syncope on January 20, 2020. Transthoracic echocardiography showed a well-defined heterogeneous, large, round mobile mass with attachments to the atrial side of the anterior mitral valve leaflet (26 × 20 mm), suggestive of an atypical myxoma (Fig. 1). In addition, the patient had a left ventricular ejection fraction (LVEF) of 55%, severe mitral stenosis, severe mitral regurgitation, mild-to-moderate tricuspid regurgitation, and a pulmonary arterial systolic pressure (PASP) of 42 mm Hg. Coronary angiography revealed normal epicardial coronary arteries. Brain computed tomography (CT) and neurologic evaluations were unremarkable.

The patient was scheduled for surgical mass excision. On January 24, the LA mass (20 × 25 mm) was removed completely, and the mitral valve was replaced with a St Jude Medical prosthetic valve given the destruction of the anterior mitral valve leaflet and chordal involvement. The histopathological examination illustrated diffuse fibrosis in the mitral valve, as well as cellular pleomorphism and high mitotic activity in the removed cardiac tumor. The evaluation of the surgical margin was rendered impossible by the fragmentation of the specimen. Postoperative echocardiography revealed that the prosthetic valve had good leaflet motion with an acceptable gradient. An LVEF of 45%, mild tricuspid regurgitation, and a PASP of 28 mm Hg were also reported. No residual mass was detected. The patient was discharged thereafter on February 3.

Seventy-nine days later, on April 22, 2020, the patient was readmitted with a 1-week history of dyspnea, palpitation, fever (body temperature = 38 °C), and a sore throat. The time in therapeutic range assessment revealed that the international normalized ratio was acceptable during the mentioned period. In the emergency department, transthoracic echocardiography demonstrated the fixation of the prosthetic mitral valve in 1 leaflet, with a mean pressure gradient of 12 mm Hg, an LVEF of 50%, moderate-to-severe tricuspid regurgitation, and a PASP of 63 mm Hg. The mitral valve fixation was evident in fluoroscopy. Laboratory examinations demonstrated a high-sensitivity C-reactive protein (hs-CRP) level of 9.9 mg/dL and a white blood cell count of 7100/μL, with a lymphocyte percentage of 18.8%.

**Fig. 2:**
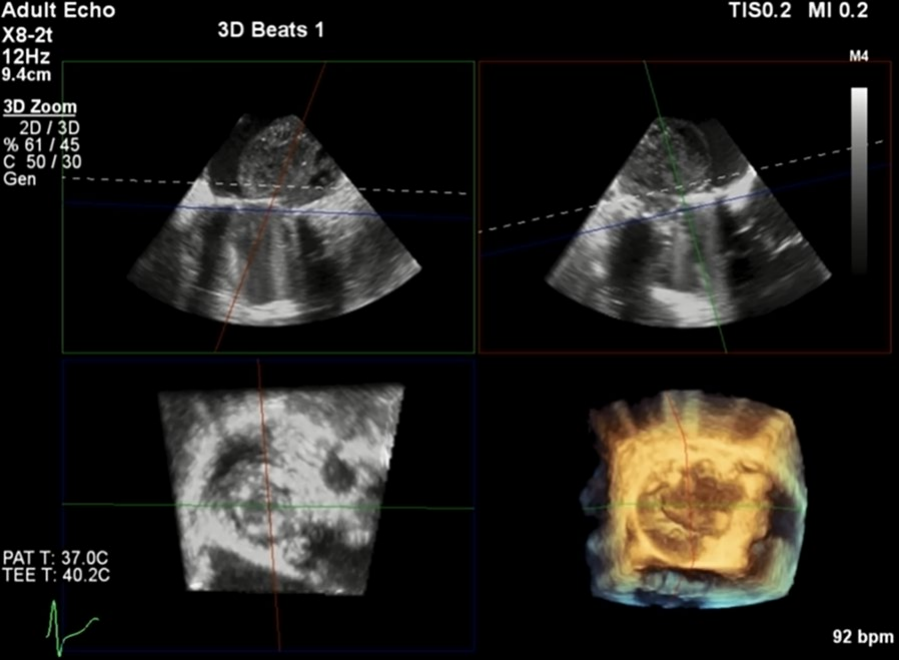
Large non-homogenous fixed mass (4 × 4 cm) on lateral left atrial wall with restricted mitral valve motion (mid-esophageal view and three-dimensional image in transesophageal echocardiography)

In the COVID-19 pandemic era, our focus was diverted to the thrombotic event of the prosthetic valve in the context of coronavirus infection, and we performed a spiral chest CT scan to exclude lung involvement. No evidence of COVID-19, except for a mild pleural effusion, was detected in the chest CT. Hence, empiric antiviral treatment with hydroxychloroquine and azithromycin was initiated with respect to the patient’s symptoms and high hs-CRP. The blood culture examination result was negative. Transesophageal echocardiography depicted a very large nonhomogeneous LA mass (40 × 40 mm) on the lateral LA wall that seemed to be an organized thrombus with restricted mitral valve leaflet motion (Fig. 2). The reverse transcription-polymerase chain reaction (RT-PCR) test was negative for COVID-19. Believing that the mass was a thrombus, we scheduled the patient for the surgical removal of the mass. The mass was surgically resected on April 27, and its gross appearance was not compatible with that of a thrombus or a pannus. Postoperative echocardiography on May 2 revealed an LVEF of 40% with acceptable motion of the prosthetic mitral valve without a visible residual mass. The microscopic evaluation of the LA mass sections revealed a neoplastic tissue composed of cells with vesicular nuclei, moderate pleomorphism, and moderate amounts of clear-to-eosinophilic cytoplasm. Some of the mentioned cells exhibited uni/multi vacuolated cytoplasm (lipoblast-like cells). A few multinucleated tumor giant cells and some inflammatory cells were also seen. The stroma showed areas of fibrosis, myxoid changes, and necrosis. Further assessment illustrated a high-grade sarcoma, a high mitotic count (8–9/10 high-power fields), necrosis below 50% of the tumoral lesion, and histologic Grade II according to the French Federation of Cancer Centers Sarcoma Group. The size and margin of the tumor could not be assessed due to the fragmentation of the specimen. Based on the immunohistochemical (IHC) staining of the specimen, a dedifferentiated liposarcoma with a high-grade sarcoma and smooth muscle differentiation topped our differential diagnosis list. The IHC findings disclosed that smooth muscle actin (SMA) and mouse double minute 2 (MDM2) markers were positive in the majority of the tumor cells; additionally, high-molecular-weight caldesmon (h-caldesmon) and Friend leukemia insertion-1 (Fli-1) markers were positive in some tumor cells. Consequently, the IHC study established the diagnosis of liposarcomas. Once the primary origin of the tumor was determined, the patient was referred to the oncology department.

## Discussion and conclusions

A dedifferentiated liposarcoma is a non-lipomatous malignancy with high cellularity and a considerably more aggressive phenotype, elevating the risk of systemic metastases and local recurrences [7]. In this case, the patient experienced an unusual in situ recurrence of the sarcomas within only 3 months after complete resection.

The 5-year survival rate for malignant cardiac tumors following treatment is about 30%, and the recurrence rate for liposarcomas is reported to be about 40% of the cases even up to 14 years after the initial surgical resection [3, 7]. In primary cardiac dedifferentiated liposarcomas, most cases are younger than 45 years old, and no sex predilection has been seen. The clinical features are generally related to the site of the tumor and the extent of infiltration [8, 9]. The most frequent symptoms in malignant cardiac tumors are dyspnea and chest pain, followed by fever with a prevalence rate of 9% [4, 10]. In the present case, the puzzling finding was an LA mass that had developed within 79 days after primary surgical resection and resulted in dyspnea. It was crucial to differentiate valvular thrombi from other possibilities such as remnants or recurrent tumors. The absence of constitutional symptoms weighed against a recurrent malignant tumor.

If the patient has a satisfactory performance status, the surgical resection of the intracavitary mass should be done. Even though cardiac tumors seem to pose a challenge to cardiac surgeons, complete surgical resection combined with adjuvant chemotherapy and/or local radiotherapy is required to lower the risk of local recurrences and systemic metastases [3, 10]. It is worthy of note that due to heart failure, which is possible in the early course of left-sided masses, neoadjuvant chemotherapy is generally contraindicated [11]. The recurrence of primary cardiac sarcomas is a common phenomenon, and close surveillance with an oncologist is essential. The present case was notable for the short interval to recurrence. The completeness of resection is a helpful feature in disease-free survival. Further, without adjuvant chemotherapy, the recurrence rate of cardiac sarcomas increases.

The concerns around the COVID-19 pandemic can give rise to misdiagnosis in cardiovascular disorders with COVID-19 infection. In the present case, the attempt to be alert in the diagnosis and the empiric treatment of COVID-19 resulted in the misdiagnosis. Even though the CT scan and RT-PCR results showed no evidence in favor of COVID-19, the patient’s symptoms and transesophageal echocardiography results were deemed related to COVID-19–induced thrombosis on the prosthetic valve. Vigilance is mandated in the diagnosis of COVID-19, but other possible disorders should not be ignored.

## Data Availability

The data underlying this article will be shared on reasonable request to the corresponding author.

## Abbreviations

AMVL: Anterior mitral valve leaflet
COVID-19: Coronavirus disease 2019
CT: Computed tomography
CVD: Cardiovascular diseases
DDLPS: Dedifferentiated Liposarcoma
DOE: Dyspnea on exertion
EF: Ejection fraction
hs-CRP: High sensitive C-reactive protein
IHC: Immunohistochemical
LA: Left atrial
LV: Left ventricle
PASP: Pulmonary artery systolic pressure
RT-PCR: Reverse transcription-polymerase chain reaction
TEE: Transesophageal echocardiography
TR: Tricuspid regurgitation
TTE: Transthoracic echocardiography
